# Association Between Conflicts of Interest and Authors’ Positions on Harms of Varenicline: a Cross-Sectional Analysis

**DOI:** 10.1007/s11606-021-06915-1

**Published:** 2021-05-26

**Authors:** Alice Fabbri, Camilla Hansen Nejstgaard, Quinn Grundy, Lisa Bero, Adam G. Dunn, Annim Mohammad, Barbara Mintzes

**Affiliations:** 1 Centre for Evidence-Based Medicine Odense (CEBMO), University of Southern Denmark and Odense University Hospital, Odense, Denmark; 2 Tobacco Control Research Group, Department for Health, University of Bath, Bath, UK; 3 Centre for Evidence-Based Medicine Odense (CEBMO) and Cochrane Denmark, Department of Clinical Research, University of Southern Denmark, Odense, Denmark; 4 Open Patient data Explorative Network (OPEN), Odense University Hospital, Odense, Denmark; 5 Lawrence S. Bloomberg Faculty of Nursing, University of Toronto, Toronto, Canada; 6 Charles Perkins Centre and School of Pharmacy, Faculty of Medicine and Health, The University of Sydney, Sydney, Australia; 7 School of Medicine and Colorado School of Public Health, University of Colorado Anschutz Medical Campus, University of Colorado Center for Bioethics and Humanities, Denver, CO USA; 8 School of Medical Sciences, Faculty of Medicine and Health, The University of Sydney, Sydney, Australia

**Keywords:** conflict of interest, pharmaceutical industry, drug safety

## Abstract

**Background:**

Few studies have investigated the relationship between industry funding/conflicts of interest and authors’ positions in opinion pieces on drug safety. Harmful effects of varenicline, a treatment for smoking cessation, have been highly contested.

**Objective:**

To examine the association between pharmaceutical industry funding/authors’ financial conflicts of interest and position on varenicline in opinion articles, especially in relation to the minimization of harms; to assess whether opinion pieces on drug safety issues written by authors with conflicts of interest are more frequently cited in the news or social media.

**Design:**

Cross-sectional analysis.

**Participants:**

English language opinion pieces and narrative reviews about varenicline published between May 2006 and February 2019.

**Main Measures:**

Odds ratios and 95% confidence intervals; the Mann-Whitney two-sample statistic was used to test for differences in Altmetric scores, a measure of media attention.

**Key Results:**

Of the 221 included articles, 30.3% (67) disclosed the funding source and 62.9% (139) disclosed authors’ conflicts of interest. Authors of opinion pieces on varenicline who reported financial ties to the pharmaceutical industry (as a conflict of interest or funding source) were more likely to minimise the cardiovascular and psychiatric risk of varenicline compared to those without conflicts of interest or industry funding (OR: 4.00; 95% CI: 1.32 to 12.16 for cardiovascular risk; OR: 8.51; 95% CI: 3.79 to 19.11 for psychiatric risk). These associations persisted in sensitivity analyses. No statistically significant difference in Altmetric score was found between articles with (mean 15.83, median 3) and without (mean 11.90, median 1) conflicts of interest, indicating similar media attention (p-value=0.11).

**Conclusions:**

We found that authors with financial ties to drug companies were more likely to publish opinion pieces that minimised harms of varenicline. These results raise questions about journals’ editorial policies to accept reviews of treatments from authors with financial relationships with manufacturers.

**Supplementary Information:**

The online version contains supplementary material available at 10.1007/s11606-021-06915-1.

## BACKGROUND

There is substantial evidence that pharmaceutical industry sponsorship of primary research is associated with outcomes more likely to favour the sponsor’s product than independent research.^1^ There have been fewer studies on how industry funding and conflicts of interest relate to authors’ positions in opinion pieces such as commentaries and editorials.^2–5^

How information is communicated in opinion pieces can be influenced by commercial interests. Pharmaceutical companies often hire medical education and communication companies to ‘ghost write’ and ‘ghost manage’ favourable articles that are published in scientific journals under the names of academic authors.^6^ These academic authors are influential experts in their field, referred to as ‘key opinion leaders’, whose views are favourable to the company’s position,^7^ and are often hired as consultants, advisors or speakers to promote these positions.^8^

A cross-sectional study of biomedical articles published in 2018 found that a significantly higher proportion of commentaries, editorials and narrative reviews included authors with conflicts of interest than primary research articles.^9^ This study also found that articles with authors with conflicts of interest received more media attention compared with other articles, which could amplify the impact of their findings.^9^

Debates about drug safety provide an opportunity to explore the association between financial conflicts of interest and authors’ positions in opinion pieces. The safety of certain drugs has been subject to scientific controversy, with authors presenting diametrically opposite views.^2^ Drug safety is also important to commercial stakeholders as safety concerns and regulatory scrutiny can negatively impact product sales.

Varenicline was approved by the US Food and Drug Administration (FDA) on 10 May 2006 for smoking cessation. Following approval, varenicline was associated with numerous safety concerns including psychiatric effects, cardiovascular effects, seizures and interactions with alcohol. In the past decade, there has been considerable debate about the use and safety of this drug: some authors recommend it as an effective and safe medication for smoking cessation,^10, 11^ whereas others have raised strong concerns about its safety.^12, 13^ In 2016, based on a review of a large clinical trial conducted by drug manufacturers at the FDA’s request, the FDA determined that there was a lower risk of serious side effects related to mood, behaviour or thinking with varenicline than previously suspected and removed a *Boxed Warning* for serious psychiatric side effects that had been added in 2009.^14^ However, drug harms are difficult to detect in RCTs if they have low event rates or occur in population groups not included in the RCTs. Concerns were raised at the FDA expert advisory committee hearing that this trial was underpowered to detect differences in serious adverse event rates, and that the full evidence on harm, including observational studies and case reports, should be considered.^15^

The aim of this study was to examine whether an association exists between authors’ financial relationships with industry and their positions on varenicline in opinion articles, especially in relation to the characterisation of harms mentioned in FDA safety advisories. We were also interested in how scientific information on harms of varenicline reaches the public. As a secondary objective, we assessed whether opinion pieces on drug safety with authors with conflicts of interest are more frequently cited or shared in the news or social media.

## METHODS

We conducted a cross-sectional analysis of all relevant opinion pieces published on varenicline since 2006.

### Search Strategy

On 13 February 2019, we conducted a literature search in PubMed and Embase for narrative reviews and opinion pieces about varenicline. We defined narrative reviews as syntheses based on a non-systematic search of the literature and opinion pieces as articles that are not original research. They included letters, commentaries, viewpoints, perspectives, editorials and drug bulletin articles (namely, articles that provide an evaluation of a medicine and sometimes also practical advice on its use). The search started from the date of the FDA drug approval in May 2006. The search strategy (Supplementary File 1) was developed with the help of a librarian.

### Identification of the Articles

One investigator carried out initial screening of titles and excluded irrelevant entries. Two investigators then independently screened the full text records. We included opinion pieces, as defined above, that mentioned at least one health outcome of varenicline use, either beneficial or harmful. Effectiveness for quitting smoking was considered a beneficial health outcome. We also included articles that commented on the methods of a study assessing benefits/harms of varenicline. Only English language articles were included.

We excluded original research, systematic reviews (defined as an appraisal and synthesis of all relevant studies on a particular topic that uses a rigorous and reproducible methodology), guidelines, conference abstracts, case reports, articles that mentioned indications but no health outcomes, articles discussing unapproved uses only (e.g. indications other than smoking cessation), articles on effects in animals only and articles without varenicline in the title and with fewer than 10 lines of text on varenicline.

### Data Extraction

We created a structured coding questionnaire (Supplementary File 2). From each included article, we extracted information using Redcap,^16^ a secure web-based data collection application.

We assessed whether any of the harms listed in the FDA safety advisories were mentioned (psychiatric effects, cardiovascular effects, seizures and alcohol interaction). Each safety concern discussed by the author was assessed separately by the coders. Authors’ positions were classified as follows: (1) stated that there is an important safety risk associated with the use of varenicline, (2) minimised the risk of harm (e.g. varenicline is safe, safety warnings are inconsistent with evidence from several sources), (3) stated there was insufficient information or (4) was unclear.

We also assessed authors’ overall position on varenicline. This was classified as positive (e.g. benefit outweighs risks, emphasises safety, minimises harms), negative (e.g. risks outweigh benefit, emphasises concerns about safety, downplays benefit), unclear (this included articles that did not express a clear positive or negative position, e.g. neutral articles).

Finally, we noted funding sources for the article and extracted information on authors’ conflicts of interest. Presence of pharmaceutical industry employees amongst the authors was counted as a funding source. We defined a conflict of interest as a declaration of financial ties between pharmaceutical or tobacco companies and at least one author. We then classified funding sources and conflicts of interest by whether companies were as follows: (1) varenicline manufacturer (Pfizer) or (2) other drug manufacturers.

We created a written manual with instructions for coders. Four rounds of pilot testing the data extraction tool preceded the data collection. During the pilot, all coders assessed the same 18 articles. Where there were differences in interpretation between coders, we revised both the data extraction tool and the written manual to improve their clarity. Two coders then independently extracted data from all articles (total of four coders, working in pairs). Discrepancies were resolved by consensus. If coders could not reach agreement, a third assessor adjudicated.

### Statistical Analysis

Summary statistics were used to describe characteristics of included articles, frequencies and types of authors’ conflicts of interest, and authors’ positions.

Odds ratios and 95% confidence intervals were calculated as a measure of the strength of association between pharmaceutical industry funding and/or authors’ conflicts of interest and position on safety concerns. Each safety concern mentioned in the FDA safety advisories was analysed separately. We compared articles that minimised risks to articles with all other positions, including positions stating that there is an important safety risk, positions stating insufficient information and unclear positions.

Odds ratios and 95% confidence intervals were calculated to explore the association between pharmaceutical industry funding and/or authors’ conflicts of interest and overall positions on varenicline. We compared articles with positive positions about varenicline’s role in smoking cessation to all other articles, including articles with negative positions, unclear positions and articles in which authors explicitly stated that not enough information was available to make a judgment.

For all primary analyses, we defined articles with conflicts of interest to be those that had the following: (1) at least one author disclosing ties with pharmaceutical companies, (2) at least one author disclosing ties with pharmaceutical and tobacco companies, (3) study funding from pharmaceutical companies or (4) combined study funding from pharmaceutical companies and other organisations. We compared this group to articles that were missing a conflict of interest/funding disclosure (no-disclosure) or declared ‘no conflicts of interest’ related to pharmaceutical or tobacco companies and had received no funding from such companies. One article declared no conflicts of interest but received funding from other industries (not pharmaceutical or tobacco). This was included in the ‘no conflicts of interest group’.

We undertook the following pre-specified sensitivity analyses:

- We excluded articles with no conflict of interest or funding disclosure.
- We included only articles funded by the manufacturer of varenicline (Pfizer) or written by authors with financial ties with Pfizer.
- We excluded the unclear articles and those that explicitly stated there was insufficient information. This allowed us to compare the articles that minimised risks to those stating that there is an important safety risk. Similarly, for overall position, we compared articles with positive positions about varenicline’s role in smoking cessation to articles with negative positions.

In two additional post hoc sensitivity analyses for cardiovascular and psychiatric safety concerns, we performed adjusted analyses accounting for articles published before or after regulatory action. For cardiovascular safety concerns, we compared articles published from 2012 onwards versus earlier articles, in order to explore the impact of the first FDA advisory on cardiovascular risks in June 2011.^17^ For psychiatric safety concerns, since only two of the included articles were published before the first psychiatric safety advisory in 2007, we compared articles published from 2017 onwards versus earlier articles, in order to explore the impact of the FDA removal of the Boxed Warning on varenicline’s psychiatric risks in December 2016.^14^

We also carried out a post hoc subgroup analysis stratifying our primary analyses on cardiovascular safety concerns, psychiatric safety concerns and overall position on varenicline by type of article (i.e. letters versus other articles).

To analyse whether articles written by authors with conflicts of interest are more frequently cited or shared in the news or social media (secondary objective), we automatically retrieved Altmetric scores using the articles’ DOI. We excluded letters from this analysis as they are unlikely to be considered newsworthy. We estimated mean and median Altmetric score for articles with and without conflicts of interest using the same definitions as our primary analyses. We then used Mann-Whitney two-sample statistic to test for a difference between the two groups. We repeated the analysis stratified on publication year to account for differences in Altmetric scores over time.

All analyses were conducted in STATA (Version 16.0)

## RESULTS

**Figure 1:**
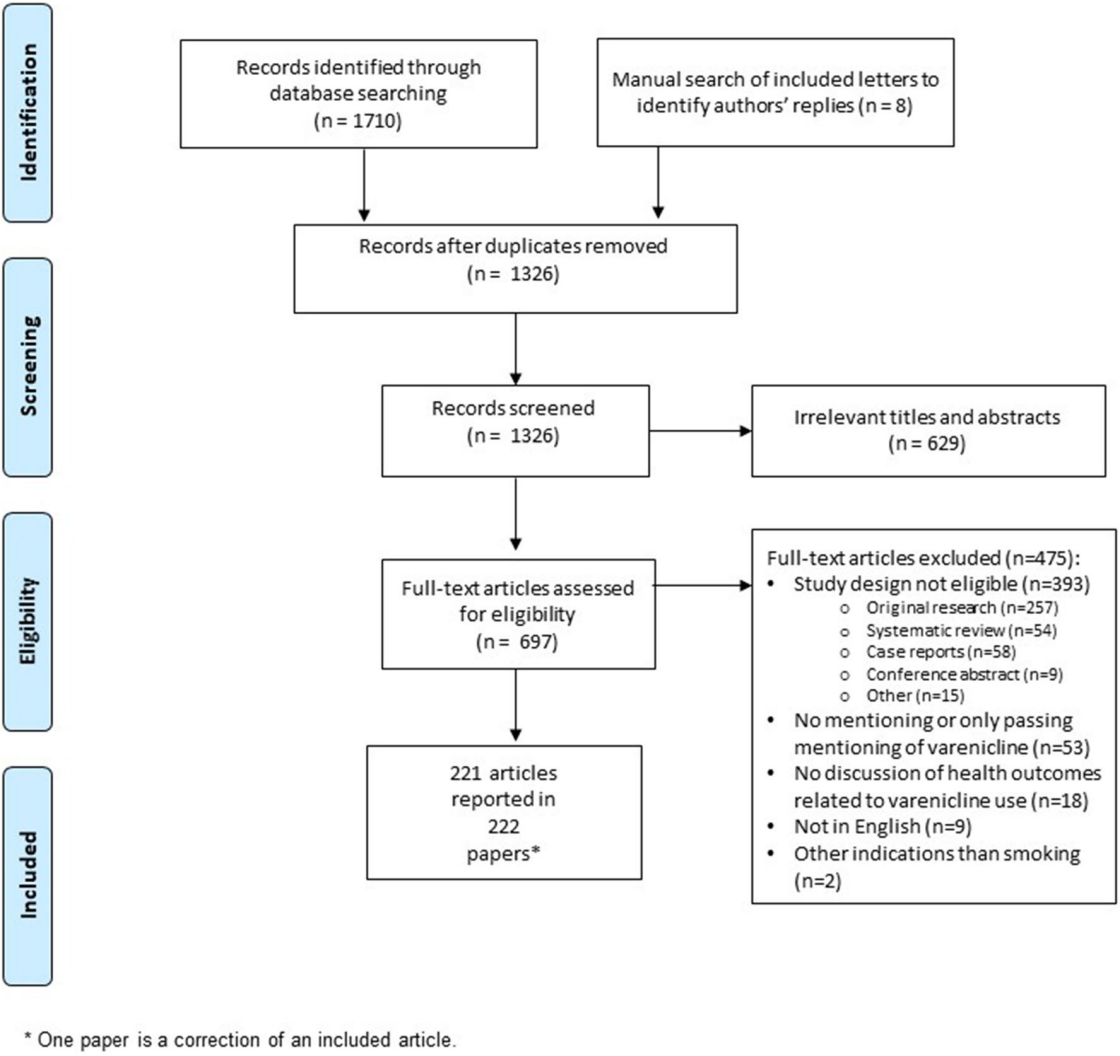
PRISMA flow diagram of included articles.

We screened 1326 articles and 221 articles, described in 222 papers (including one correction), met the inclusion criteria (Fig. 1). We used the denominator of 221 for all analyses to avoid double-counting the corrected article. Authors’ replies to letters were not captured consistently in the literature search, and we therefore conducted a supplementary manual search (*n*=8 added). For 13 articles (5.9%), coders’ judgments were resolved via adjudication by a third assessor. Of the 221 included articles, 34.0% (75/221) were narrative reviews; 31.7% (70/221) were editorials, commentaries or viewpoints; 24.0% (53/221) were letters; 8.6% (19/221) were bulletin articles; and 1.8% (4/221) were other types of opinion pieces. Most of the articles (83.2%, 184/221) focused on the general population while 16.7% (37/221) focused on specific subgroups (e.g. smokers with mental illness or with cardiovascular disease). A full bibliography of included articles is available as Supplementary File 3.

### Disclosure of Funding Source and Financial Conflicts of Interest

Funding source was disclosed in 30.3% (67/221) of the articles. Authors disclosed pharmaceutical industry funding (alone or mixed with other funding sources) in 6.8% (15/221) of articles; 14 of these 15 were funded by Pfizer. One article reported tobacco industry funding (mixed with other sources). Thirteen articles (13/221, 5.9%) stated that no specific funding was received for the development of the manuscript.

A conflict of interest statement was present in 62.9% (139/221) of the articles and 33.0% (73/221) disclosed author financial ties with pharmaceutical companies. In one of these 73 articles, financial ties with the tobacco industry were also disclosed. The authors had ties with Pfizer (solely or with other companies) in 29.9% (66/221) of sampled articles. In another 29.9% (66/221), the authors disclosed that they had no conflicts of interest involving either pharmaceutical or tobacco companies.

### Classification of Authors’ Positions

Efficacy was mentioned in 72.4% (160/221) of the articles. Authors’ positions were that varenicline is effective for smoking cessation in 57.5% (127/221) of the articles, that there is not enough information available on efficacy (especially in specific subgroups of patients) in 7.2% (16/221), that efficacy is limited in some ways (e.g. the beneficial effect is not maintained in the long term) in 6.3% (14/221) and that it is not effective (e.g. it does not increase successful quitting) in 1.4% (3/221). Varenicline’s brand names were mentioned in 25.8% (57/221) articles.

In terms of the harms mentioned in the FDA safety advisories, psychiatric and cardiovascular risks were mentioned in 59% (131/221) and 30% (67/221) of the articles, respectively. Seizures were mentioned in 5% (10/221) and alcohol interactions in 4% (9/221) of the articles (Table 1). Other adverse effects (e.g. nausea, headache) were described in 42.1% (93/221) of the articles. The authors’ overall position on varenicline was judged to be positive in 55.5% (122/221), negative in 13.6% (30/221) and unclear in 23.1% (51/221); in 8.1% (18/221) of articles, the authors stated there was not enough information to draw conclusions.

**Table 1:** Authors’ Position on Risks Associated with Varenicline Use

	Psychiatric (n=131)	Cardiovascular (n=67)	Seizures (n=10)	Alcohol interactions (n=9)
	N (%)	N (%)	N (%)	N (%)
States there is an important safety risk	47 (36)	17 (25)	6 (60)	4 (44)
Minimises the risk	49 (37)	34 (51)	2 (20)	0
Insufficient information	18 (14)	6 (9)	0	0
Unclear position	17 (13)	10 (15)	2 (20)	5 (56)

### Funding Source/Conflicts of Interest and Authors’ Positions

We planned to separately analyse each of the four safety concerns mentioned in the FDA advisories, but for seizures (10/221) and alcohol interactions (9/221) there were too few articles to conduct a separate analysis. Tables 2 and 3 describe results for cardiovascular and psychiatric risk.

**Table 2:** Authors’ Financial Conflict of Interest Versus Positions on Safety Concerns and Overall Position on Varenicline

	**Cardiovascular safety concerns** **(n=67)**		
**Author position**	**COI**^**a**^ (n=22) N (%)	**No COI**^**b**^ (n=22) N (%)	**No disclosure** (n=23) N (%)
States there is an important safety risk	2 (9)	7 (32)	8 (35)
Minimised risk	16 (73)	9 (41)	9 (39)
Insufficient information	1 (5)	3 (14)	2 (9)
Unclear	3 (14)	3 (14)	4 (17)
	**Psychiatric safety concerns** **(n=131)**		
	**COI**^**a**^ (n=49) N (%)	**No COI**^**b**^ (n=49) N (%)	**No disclosure** (n=33) N (%)
States there is an important safety risk	5 (10)	24 (49)	18 (55)
Minimised risk	33 (67)	11 (22)	5 (15)
Insufficient information	6 (12)	5 (10)	7 (21)
Unclear	5 (10)	9 (18)	3 (9)
	**Overall position on varenicline** **(n=221)**		
	**COI**^**a**^ (n=80) N (%)	**No COI**^**b**^ (n=73) N (%)	**No disclosure** (n=68) N (%)
Negative	1 (1)	17 (23)	12 (18)
Positive	67 (84)	24 (33)	31 (46)
Insufficient information	4 (5)	9 (12)	5 (7)
Unclear	8 (10)	23 (32)	20 (29)

*COI* conflicts of interest

^a^Includes articles disclosing pharmaceutical industry funding (alone or with other organisations), authors’ conflicts of interest with pharmaceutical companies or both

^b^Includes articles with no conflicts of interest and no pharmaceutical industry funding

**Table 3:** Association Between Financial Conflicts of Interest and Author’s Position on Cardiovascular and Psychiatric Safety Concerns

	Cardiovascular safety concerns			Psychiatric safety concerns		
	Minimised risk	States there is an important safety risk/insufficient information/unclear	OR (95% CI)	Minimised risk	States there is an important safety risk/insufficient information/unclear	OR (95% CI)
Author COI	N (%)	N (%)		N (%)	N (%)	
Primary analysis						
COI*	16 (73)	6 (27)	4.00 (1.32–12.16)	33 (67)	16 (33)	8.51 (3.79–19.11)
No COI**	18 (40)	27 (60)	–	16 (20)	66 (80)	–
Sensitivity analysis excluding articles with no COI or funding disclosure						
COI*	16 (73)	6 (27)	3.85 (1.09–13.66)	33 (67)	16 (33)	7.13 (2.90–17.49)
No COI***	9 (41)	13 (59)	–	11 (22)	38 (78)	–

*COI* conflicts of interest, *OR* odds ratio, *CI* confidence interval

*Includes articles disclosing pharmaceutical industry funding (alone or with other organisations), authors’ conflicts of interest with pharmaceutical companies or both

**Includes articles with no COI, no pharmaceutical industry funding and no COI disclosure

***Includes articles with no COI and no pharmaceutical industry funding

We had initially planned to analyse funding or conflicts of interest of one or more authors related to Pfizer and other pharmaceutical companies separately but did not perform this sensitivity analysis as funding/conflicts of interest with Pfizer dominated the sample and did not allow for comparison. Of the 67 articles that mentioned cardiovascular safety concerns, 22 disclosed pharmaceutical industry funding or conflicts of interest, all of which were related to Pfizer (alone or with other companies). Of the 131 articles that mentioned psychiatric concerns, 49 disclosed industry funding or conflicts of interest, 95.9% (47/49) of which were related to Pfizer (alone or with other companies). When we assessed authors’ overall position on varenicline, 80/221 articles disclosed industry funding or conflicts of interest, 90% (72/80) of which were related to Pfizer (alone or with other companies).

### Analyses on Cardiovascular Safety Concerns

Authors of opinion pieces on varenicline were more likely to minimise the risk when they had conflicts of interest with the pharmaceutical industry or the article was industry-funded (OR: 4.00; 95% CI: 1.32–12.16). We conducted three sensitivity analyses and found similar results (Table 3; Supplementary File 4, Tables S1 and S2). One subgroup analysis found a lower OR for letters (OR: 2.29; 95% CI: 0.32–16.51) than other types of articles (OR: 5.18; 95% CI: 1.34–20.06), though with overlapping 95% CI (Supplementary File 4, Table S4).

### Analyses on Psychiatric Safety Concerns

Authors were more likely to minimise psychiatric risks if they had conflicts of interest or the article was funded by the pharmaceutical industry (OR: 8.51; 95% CI: 3.79–19.11). The two pre-specified sensitivity analyses confirmed this association (Table 3; Supplementary File 4, Table S1). When we adjusted for publication year (articles published before and after the FDA removal of the Boxed Warning for varenicline’s psychiatric risks), we found similar results as our primary analysis (OR: 10.52; 95% CI: 4.40–25.16) (Supplementary File 4, Table S3). One sub-group analysis found a higher OR for letters (OR: 24.00; 95% CI: 1.95–295.06) than for other types of articles (OR: 7.14; 95% CI: 2.98–17.11; Supplementary File 4, Table S4).

### Analyses on Overall Position on Varenicline

In an analysis of authors’ overall position in relation to conflicts of interest (Table 4), opinion pieces were more likely to be positive about varenicline’s role in smoking cessation if the authors had conflicts of interest or the article was pharmaceutical industry-funded (OR: 8.06; 95% CI: 4.07–15.96) as compared with other articles.

**Table 4:** Association Between Financial Conflicts of Interest and Author’s Overall Position on Varenicline

	Overall position on varenicline					
	Negative/ unclear/ insufficient information		Positive		OR	95% CI
Author COI	N	%	N	%		
Primary analysis						
COI*	13	16	67	84	8.06	4.07–15.96
No COI**	86	61	55	39	–	
Sensitivity analysis excluding articles with no COI or funding disclosure						
COI*	13	16	67	84	10.53	4.88–22.70
No COI***	49	67	24	33	–	

*COI* conflicts of interest, *OR* odds ratio, *CI*: confidence interval

*Includes articles disclosing pharmaceutical industry funding (alone or with other organisations), authors’ conflicts of interest with pharmaceutical companies or both

**Includes articles with no COI, no pharmaceutical industry funding and no COI disclosure

***Includes articles with no COI and no pharmaceutical industry funding

The association found in our primary analysis was confirmed in two sensitivity analyses. (Table 4; Supplementary File 4, Table S1) One subgroup analysis found a higher OR for letters (OR: 12.00; 95% CI: 3.07–46.88) than other types of articles (OR: 7.86; 95% CI: 3.41–18.11; Supplementary File 4, Table S4).

### Analyses on Altmetric Scores

Our secondary objective was to assess whether opinion pieces on varenicline funded by industry or written by authors with conflicts of interest are more frequently discussed or shared in the news/social media. Altmetric scores were only available for articles published from 2011 onwards (n=133/221, 60.2%). After excluding letters, our final sample was 100 articles. We found no statistically significant difference in Altmetric score between articles with and without conflicts of interest (articles with conflicts, mean score 15.83, median 3; articles without conflicts, mean score 11.90, median 1; p value: 0.11). Stratified results for publication year failed to indicate any association between conflicts of interest and Altmetric score (Supplementary File 4, Table S5).

## DISCUSSION

We found a strong association between authors’ financial conflicts of interest, minimization of safety concerns and favourable positions on varenicline’s role in smoking cessation. We observed a similar pattern for cardiovascular and psychiatric risks and multiple sensitivity analyses confirmed this association. However, our hypothesis that opinion pieces written by authors with conflicts of interest would be more frequently cited or shared in the news or social media was not confirmed.

Previous studies have found an association between authors’ views on safety concerns and conflicts of interest.^2^ For example, Wang et al. found that authors who dismissed or minimised cardiac risks of rosiglitazone were more likely to have financial conflicts of interest.^2^ This may be because industry financing affects how the benefits and harms of companies’ products are perceived. Alternatively, companies may be more likely to provide funding to authors with favourable views on a medicine. Citation bias is another mechanism that might be at play, whereby favourable messages are built by selectively citing or ignoring evidence. For example, a study of reviews of influenza treatments found that authors’ conclusions could be predicted by analysing which references they cited and which they omitted.^18^

The safety concerns addressed in this study were highlighted in regulatory safety advisories aiming to protect patient health and contribute to safer prescribing. Commentaries that minimise these risks may have an impact on patient care. It is also interesting to note that the association between financial relationships with industry and position on psychiatric risks persisted even after the FDA reversed its position on psychiatric risks. In December 2016, new evidence led the FDA to withdraw a black box warning of varenicline’s psychiatric risks and to state that the risk, although still present, was weaker than expected.^14^ The revised FDA regulatory decision was likely to have influenced authors’ assessments. However, when we analysed the relationship between conflicts of interest and authors’ position on psychiatric risks in articles published before and after FDA reversal, we found similar results as in our primary analysis.

In the included articles, funding source was rarely disclosed (30.3%). This was expected since opinion pieces rarely have specific funding. More surprising was the lack of reporting of authors’ conflicts of interest in more than 30% of the included articles. We do not know whether the lack of disclosure reflects inadequate journal disclosure policies, a failure to implement policies or authors’ failure to comply. Whatever the reason, the low rate of disclosure requires continuous efforts to address this reporting gap.^19^

This study has some limitations. First, the conclusions are limited by the observational nature of the research. These findings show an association, not a cause-and-effect relationship. Second, we relied on disclosures in publications and did not perform any additional search for unreported conflicts of interest. We may therefore have underestimated the prevalence of conflicts of interest. Third, data collectors were not blinded to funding and conflict of interest disclosures as this information was in the article text. Fourth, we could not clarify whether effects of financing by varenicline’s manufacturer, Pfizer, differed from effects of financing by other companies as most conflicts of interest were with Pfizer.

## CONCLUSIONS

We found an association between authors’ financial conflicts of interest and positions that minimised safety concerns associated with varenicline use and advanced a positive view of its role in smoking cessation. These findings raise concerns about journals’ editorial policies to accept reviews and commentaries on drug treatments from authors with financing from manufacturers and support the need for independent review.

## Supplementary Information

ESM 1 (DOCX 13 kb)

ESM 2 (DOCX 18 kb)

ESM 3 (DOCX 28 kb)

ESM 4 (DOCX 22 kb)
