# Supplementary material for: Association Between Conflicts of Interest and Authors’ Positions on Harms of Varenicline: a Cross-Sectional Analysis
Source: J Gen Intern Med. 2021 May 26;37(2):290–7. doi: 10.1007/s11606-021-06915-1 (PMC8811060; doi:10.1007/s11606-021-06915-1)
Supplement: Supplementary file 1 — (DOCX 13 kb) [file 11606_2021_6915_MOESM1_ESM.docx]

**Supplementary File 1. Search strategy**

**PUBMED**

**Block 1: Varenicline**

1. Varenicline OR Chantix OR Champix

2. Varenicline (MeSH)

3. 1 OR 2

**Block 2: opinion pieces**

4. Editorial OR editorials OR essay OR essays OR commentary OR commentaries OR comment OR comments OR letter OR letters OR response OR responses OR reply OR replies OR blog OR blogs OR viewpoint OR viewpoints

5. (Narrative OR descriptive OR non-systematic OR non-systematical OR non-systematically OR nonsystematic OR nonsystematical OR nonsystematically) AND (review OR reviews OR overview OR overviews)

6. (Non systematic OR non systematical OR non systematically) AND (review OR reviews OR overview OR overviews)

7. (Expert OR experts) AND (opinion OR opinions)

8. Expert Testimony (MeSH)

9. Comment [Publication Type]

10. Editorial [Publication Type]

11. Essays [Publication Type]

12. Letter [Publication Type]

13. Blog [Publication Type]

14. 4 OR 5 OR 6 OR 7 OR 8 OR 9 OR 10 OR 11 OR 12 OR 13

**Combined searches**

15. 3 AND 14

16. Search 3, Filters: Editorial; Comment; Letter

17. 15 OR 16

18. Search 17, Filters: Publication date from 2007/05/10

**EMBASE**

**Block 1: Varenicline**

1. Varenicline OR Chantix OR Champix

2. Varenicline (Emtree)

3. 1 OR 2

**Block 2: opinion pieces**

4. Editorial OR editorials OR essay OR essays OR commentary OR commentaries OR comment OR comments OR letter OR letters OR response OR responses OR reply OR replies OR blog OR blogs OR viewpoint OR viewpoints

5. (Narrative OR descriptive OR non-systematic OR non-systematical OR non-systematically OR nonsystematic OR nonsystematical OR nonsystematically) AND (review OR reviews OR overview OR overviews)

6. (Non systematic OR non systematical OR non systematically) AND (review OR reviews OR overview OR overviews)

7. (Expert OR experts) ADJ3 (opinion OR opinions)

8. 4 OR 5 OR 6 OR 7

**Combined searches**

9. 3 AND 8

10. limit 3 to (editorial or letter or note)

11. 9 OR 10

12. limit 11 to yr=”2006-Current”
