# Supplementary material for: Association Between Conflicts of Interest and Authors’ Positions on Harms of Varenicline: a Cross-Sectional Analysis
Source: J Gen Intern Med. 2021 May 26;37(2):290–7. doi: 10.1007/s11606-021-06915-1 (PMC8811060; doi:10.1007/s11606-021-06915-1)
Supplement: Supplementary file 2 — (DOCX 18 kb) [file 11606_2021_6915_MOESM2_ESM.docx]

**Supplementary File 2. Data extraction form**

| Variable | Coding instruction |
| --- | --- |
| Title |  |
| Journal |  |
| Year of publication |  |
| Name of first author |  |
| Type of article |  |
| Journal |  |
| Is the article focused on | 1. General population 2. One or more patient subgroups (please specify) |

**Assessing authors’ position/view**

| Does the article mention specific brand names? | 1=Yes  2=No |
| --- | --- |
| What is the authors’ position on the efficacy of varenicline for smoking cessation? | 1=Effective  2=Ineffective  3= States there is not enough information  4= Does not mention  5=States that efficacy is limited in some way (e.g. the beneficial effect is not maintained in the long term or the study explicitly says that varenicline is effective in a certain patient population but not in another)  Note: efficacy or lack of effect should be stated explicitly. |
| Do the authors present any data (numbers) on effectiveness? | 1=Yes  2=No |
| Please copy the relevant text on efficacy or lack of effect |  |
| Do the authors specifically mention any of the safety concerns listed in the FDA safety advisories? | 1=Psychiatric  2=Cardiovascular  3=Seizures  4=Alcohol interaction (Decreased tolerance to alcohol including increased drunkenness, unusual or aggressive behaviour)  5=None of the above |
| For each of the safety concerns mentioned above, answer the following three questions  (if the safety concern is mentioned in the article) | |
| Do the authors present any data (numbers) on [safety concern]? | 1=Yes  2=No |
| What is the overall position on [safety concern]? | States there is an important safety risk  Minimises the risk  States there is insufficient information  Unclear |
| Copy and paste sections of the article that support your judgement | Note: please copy and paste the sentences that best support your judgement |
|  | |
| Are any other adverse effect mentioned?  (these might include both serious and non serious adverse effects) | 1=Yes  2=No |
| If yes, please write down the adverse effect |  |
| What is the authors’ overall position on varenicline? | Positive overall (e.g. Benefit outweighs risks, overall favours benefit, emphasizes safety, minimises harm, criticizes authors questioning safety)  Negative overall (e.g. Risks outweigh benefit, overall favours harms, emphasizes concerns about safety, downplays benefit or value of benefit, criticizes authors emphasizing the safety of the drug, stresses limits to effectiveness)   1. Explicitly states insufficient information 2. Unclear (this can also include neutral articles) |

**Assessment of funding source and COI**

| Disclosed authors’ financial conflicts of interest | 1= There is no COI disclosure  2= The authors state they have no COI with pharmaceutical companies or tobacco companies  3= Yes, there is a COI with pharmaceutical companies (excluding industry employees)  4= Yes, there is COI with tobacco companies  5= Yes, COI with both Pharma and Tobacco |
| --- | --- |
| Disclosed authors’ financial conflicts of interest | Verbatim |
| If there is a COI with the pharmaceutical industry, does at least one of the authors have a COI with the manufacturer of the drug (Pfizer)? | 1=Yes  2=No |
| Is any of the conflicted authors the first or the last author? | 1=Yes  2=No |
| Are any of the authors employees of pharmaceutical companies? | 1=Yes, of the drug manufacturer  2=Yes, of other pharmaceutical companies  3=No  4= No information |
| Are any of the authors employees of tobacco companies? | 1=Yes  2=No  3= No information |
| Is there a separate funding source listed for the article? | 1= Not disclosed  2= States there is no specific funding  3= Pharmaceutical industry  4= Tobacco industry  5=Other industry funding sources  6=Other public or not-for-profit funding sources  Note for the coders: if authors say they are employees of the drug manufacturer or tobacco companies, we will consider this a form of study funding (salary) rather than authors’ COI. |
| Funding sources (if disclosed) | Verbatim |
| If the funder is the pharmaceutical industry | 1=The manufacturer of the drug  2=Other pharmaceutical companies |
