# Supplementary material for: Association Between Conflicts of Interest and Authors’ Positions on Harms of Varenicline: a Cross-Sectional Analysis
Source: J Gen Intern Med. 2021 May 26;37(2):290–7. doi: 10.1007/s11606-021-06915-1 (PMC8811060; doi:10.1007/s11606-021-06915-1)
Supplement: Supplementary file 3 — (DOCX 28 kb) [file 11606_2021_6915_MOESM3_ESM.docx]

**Supplementary File 3. Included articles**

1. Allenby CE, Boylan KA, Lerman C, Falcone M. Precision Medicine for Tobacco Dependence: Development and Validation of the Nicotine Metabolite Ratio. Journal of Neuroimmune Pharmacology. 2016;11(3):471-83.
2. Alper BS. Varenicline: Quantifying the risk. CMAJ. 2011;183(12):1405.
3. Anaizi N. Will the doctor stop smoking?! Libyan Journal of Medicine. 2009;4(1):1-3.
4. Annamalai A, Singh N, O'Malley SS. Smoking use and cessation among people with serious mental illness. Yale Journal of Biology and Medicine. 2015;88(3):271-7.
5. Varenicline effective for smoking cessation. Journal of Family Practice. 2006;55(10):848-9.
6. Varenicline (Chantix) for tobacco dependence. Medical Letter on Drugs and Therapeutics. 2006;48(1241-1242):66-8.
7. Varenicline: Depression and suicide. Prescrire International. 2008;17(95):113.
8. Varenicline: A british review. Prescrire International. 2008;17(97):199.
9. Varenicline (Chantix) warnings: Risk versus benefit. Medical Letter on Drugs and Therapeutics. 2008;50(1290):53.
10. Varenicline: The French experience after 6 months. Prescrire International. 2009;18(99):25.
11. Smoking cessation during pregnancy. Obstetrics and Gynecology. 2010;116(5):1241-4.
12. Safety of smoking cessation drugs. Obstetrics and Gynecology. 2010;115(1):176.
13. Varenicline and cardiovascular events? Drug and Therapeutics Bulletin. 2011;49(9):100.
14. More evidence that varenicline harms the heart. The Harvard mental health letter / from Harvard Medical School. 2011;28(4):7.
15. In brief: Cardiovascular safety of varenicline (Chantix). The Medical letter on drugs and therapeutics. 2011;53(1371):65.
16. Smoking cessation drug varenicline and risk of depression and suicidal behaviour. Drug and Therapeutics Bulletin. 2012;50(1):2-.
17. Combining interventions to help people to stop smoking. Drug and Therapeutics Bulletin. 2013;51(1):2.
18. Varenicline plus NRT better than varenicline alone for stopping smoking. Drug and Therapeutics Bulletin. 2014;52(9):100.
19. Smoking cessation pharmacotherapy and neuropsychiatric adverse effects. Drug and Therapeutics Bulletin. 2014;52(1):5.
20. Varenicline safety. Drug and Therapeutics Bulletin. 2015;53(12):137.
21. Neuropsychiatric safety of varenicline and bupropion. Drug and Therapeutics Bulletin. 2016;54(7):76-7.
22. Corrigendum to: Discovery and development of varenicline for smoking cessation (Expert Opinion on Drug Discovery, (2018), 13, 7, (671-683), 10.1080/17460441.2018.1458090). Expert Opinion on Drug Discovery. 2018;13(7):9-11.
23. Anthenelli RM. Forget the myths and help your psychiatric patients quit smoking. Current Psychiatry. 2016;15(10):23-5.
24. Antoniu SA, Trofor AC. Varenicline for smoking cessation intervention in chronic obstructive pulmonary disease. Expert Opinion on Pharmacotherapy. 2011;12(16):2595-7.
25. Ashare RL, Falcone M, Lerman C. Cognitive function during nicotine withdrawal: Implications for nicotine dependence treatment. Neuropharmacology. 2014;76(PART B):581-91.
26. Aubin H-J, Bobak A, Britton JR, Oncken C, Billing CB, Gong J, et al. Authors’ reply. Thorax. 2008;63(8):752-3.
27. Aubin H-J, Bobak A, Britton JR, Oncken C, Billing CB, Gong J, et al. Authors’ reply. Thorax. 2008;63(8):752-.
28. Aubin HJ, Rollema H, Svensson TH, Winterer G. Smoking, quitting, and psychiatric disease: A review. Neuroscience and Biobehavioral Reviews. 2012;36(1):271-84.
29. Aveyard P. The place of varenicline in smoking cessation treatment. Thorax. 2008;63(8):666-8.
30. Balbani APS, Montovani JC. Recent developments for smoking cessation and treatment of nicotine dependence. Expert Opinion on Therapeutic Patents. 2007;17(3):287-97.
31. Baraona LK, Lovelace D, Daniels JL, McDaniel L. Tobacco Harms, Nicotine Pharmacology, and Pharmacologic Tobacco Cessation Interventions for Women. Journal of Midwifery and Women's Health. 2017;62(3):253-69.
32. Barboza J. Pharmaceutical strategies for smoking cessation during pregnancy. Expert Opinion on Pharmacotherapy. 2018;19(18):2033-42.
33. Barboza JL, Patel R, Patel P, Hudmon KS. An update on the pharmacotherapeutic interventions for smoking cessation. Expert Opinion on Pharmacotherapy. 2016;17(11):1483-96.
34. Benowitz NL. Pharmacology of nicotine: Addiction, smoking-induced disease, and therapeutics. Annual Review of Pharmacology and Toxicology. 2009;49:57-71.
35. Benowitz NL, Prochaska JJ. Smoking cessation after acute myocardial infarction. Journal of the American College of Cardiology. 2013;61(5):533-5.
36. Bentz CJ. Review: Nicotine gum and varenicline, but not behavioral interventions, reduce relapse in persons who have stopped smoking - Commentary. Annals of Internal Medicine. 2009;151(2):JCI-11.
37. Bland P. Weighing up the risks and benefits of smoking cessation therapies. The Practitioner. 2013;257(1766):5.
38. Blankfield RP. Varenicline: quantifying the risk. CMAJ 2011;183(12):1404; author reply 5, 7.
39. Bonertz L. Varenicline. Canadian Pharmacists Journal. 2008;141(2):71.
40. Bozinoff N, Le Foll B. Understanding the implications of the biobehavioral basis of nicotine addiction and its impact on the efficacy of treatment. Expert Review of Respiratory Medicine. 2018;12(9):793-804.
41. Braillon A. When will French smokers be concerned by varenicline's benefit-to-risk ratio? The Lancet Respiratory Medicine. 2016;4(4):e13.
42. Braillon A. Long-term smoking cessation: from general practice to public health. International journal of epidemiology. 2018;47(4):1355.
43. Brock AJ, Takeda A, Brennan C, Walton RT. Treatment for tobacco dependence: A potential application for stratified medicine? Personalized Medicine. 2011;8(5):571-9.
44. Brophy J. ACP Journal Club. Review: Varenicline is better than placebo or bupropion, but not clearly different from the nicotine patch, for smoking abstinence. Annals of internal medicine. 2011;154(8):Jc4-.
45. Brophy JM. ACP Journal Club. Review: Varenicline increases risk for serious adverse cardiovascular events in tobacco users. Annals of internal medicine. 2011;155(8):Jc4-5.
46. Brown D, Ewigman B. Newer study shows smoking cessation aid is safe. Journal of Family Practice. 2013;62(4):174.
47. Burke MV, Hays JT, Ebbert JO. Varenicline for smoking cessation: A narrative review of efficacy, adverse effects, use in at-risk populations, and adherence. Patient Preference and Adherence. 2016;10:435-41.
48. Cantrell MA, Geraets D. Varenicline for tobacco dependence. The New England journal of medicine. 2009;360(7):730-1.
49. Caponnetto P, Russo C, Polosa R. Smoking cessation: Present status and future perspectives. Current Opinion in Pharmacology. 2012;12(3):229-37.
50. Carrozzi L, Pistelli F, Viegi G. Pharmacotherapy for smoking cessation. Therapeutic Advances in Respiratory Disease. 2008;2(5):301-17.
51. Carson KV, Usmani ZA, Robertson TA, Mysore S, Brinn MP. Smoking cessation interventions for lung cancer patients. Lung Cancer Management. 2013;2(1):61-74.
52. Cayley Jr WE. Interventions to help patients reduce or eliminate the use of smokeless tobacco. American Family Physician. 2009;80(11):1226.
53. Cerny EH, Cerny T. Vaccines against nicotine. Human Vaccines. 2009;5(4):200-5.
54. Chapman S, MacKenzie R. Can it be ethical to apply limited resources in low-income countries to ineffective, low-reach smoking cessation strategies? A reply to Bitton and Eyal. Public Health Ethics. 2012;5(1):29-37.
55. Chatterjee S. Comparing varenicline with and without nicotine replacement therapy for smoking cessation. Cardiology Review. 2014;30(6).
56. Cherry DC, Remenchik E. New and old strategies for tobacco cessation. Clinical Pulmonary Medicine. 2007;14(4):206-11.
57. Cheung KW. The role of emergency physicians in tobacco cessation and British Columbia's smoking cessation program. Canadian Journal of Emergency Medicine. 2014;16(2):89-90.
58. Chu CW, Kao CH, Chan CK. Could the nicotine receptor partial agonist varenicline moderate manic symptoms in heavy smokers with manic episodes? Australian and New Zealand Journal of Psychiatry. 2018;52(2):205.
59. Conigrave KM, Lee KSK. Smoking or Alcohol Dependence Among Indigenous Australians: Treatment May Be Needed, Not Just Education. Heart Lung and Circulation. 2012;21(10):626-31.
60. Cressman AM, Pupco A, Kim E, Koren G, Bozzo P. Smoking cessation therapy during pregnancy. Canadian Family Physician. 2012;58(5):525-7.
61. Cropsey KL, Clark CB, Hendricks PS. Varenicline for smoking reduction PriortoCessation. JAMA. 2015;313(22):2284-5.
62. Crunelle CL, Miller ML, Booij J, van den Brink W. The nicotinic acetylcholine receptor partial agonist varenicline and the treatment of drug dependence: A review. European Neuropsychopharmacology. 2010;20(2):69-79.
63. Cummings KM, Mahoney M. Current and emerging treatment approaches for tobacco dependence. Current Oncology Reports. 2006;8(6):475-83.
64. Dal Pan GJ. Commentary on "The Effectiveness of Varenicline Medication Guide for Conveying Safety Information to Patients: A REMS Assessment Survey" by Enger et al. Pharmacoepidemiology and Drug Safety. 2013;22(7):716-8.
65. Das S, Prochaska JJ. Innovative approaches to support smoking cessation for individuals with mental illness and co-occurring substance use disorders. Expert Review of Respiratory Medicine. 2017;11(10):841-50.
66. Davies NM, Taylor G, Taylor AE, Martin RM, Munafo MR, Thomas KH. Cardiovascular and neuropsychiatric risks of varenicline: Too good to be true? The Lancet Respiratory Medicine. 2015;3(12):e39-e40.
67. Davies NM, Thomas KH. The Food and Drug Administration and varenicline: should risk communication be improved? Addiction (Abingdon, England). 2017;112(4):555-8.
68. Davies SP. Still not clear that smoking cessation drugs do not cause psychiatric symptoms. BMJ (Online). 2013;347 (no pagination)(f7065).
69. De Long NE, Barra NG, Hardy DB, Holloway AC. Is it safe to use smoking cessation therapeutics during pregnancy? Expert opinion on drug safety. 2014;13(12):1721-31.
70. Dervaux A, Kanit M, Laqueille X. Efficacy of varenicline for smoking cessation. Jama. 2006;296(21):2555; author reply -6.
71. Dhelaria RK, Rothberg M. Is varenicline effectiveness declining in randomized trials? Archives of Internal Medicine. 2011;171(19):1770-1.
72. Dodd S, Arancini L, Gomez-Coronado N, Gasser R, Lubman DI, Dean OM, et al. Considerations when selecting pharmacotherapy for nicotine dependence. Expert Opinion on Pharmacotherapy. 2019;20(3):245-50.
73. Doggrell SA. Partial agonism at nicotinic receptors with varenicline--a new approach to smoking cessation. Expert opinion on pharmacotherapy. 2006;7(18):2599-603.
74. Doggrell SA. Which is the best primary medication for long-term smoking cessation - Nicotine replacement therapy, bupropion or varenicline. Expert Opinion on Pharmacotherapy. 2007;8(17):2903-15.
75. Drach L, Holbert T, Maher J, Fox V, Schubert S, Saddler LC. Integrating smoking cessation into HIV care. AIDS Patient Care and STDs. 2010;24(3):139-40.
76. Ebbert JO. Varenicline and combination nicotine replacement therapy are the most effective pharmacotherapies for treating tobacco use. Evidence-Based Medicine. 2013;18(6):212-3.
77. Ebbert JO, Fagerstrom K. Pharmacological interventions for the treatment of smokeless tobacco use. CNS Drugs. 2012;26(1):1-10.
78. Ebbert JO, Hays JT, Hurt RD. Combination pharmacotherapy for stopping smoking: What advantages does it offer? Drugs. 2010;70(6):643-50.
79. Ebbert JO, Hughes JR, West RJ. Varenicline for smoking reduction prior to cessation--reply. Jama. 2015;313(22):2285-6.
80. Elrashidi MY, Ebbert JO. Emerging drugs for the treatment of tobacco dependence: 2014 update. Expert Opinion on Emerging Drugs. 2014;19(2):243-60.
81. Escobar-Chavez JJ, Dominguez-Delgado CL, Rodriguez-Cruz IM. Targeting nicotine addiction: The possibility of a therapeutic vaccine. Drug Design, Development and Therapy. 2011;5:211-24.
82. Etter JF, Lukas RJ, Benowitz NL, West R, Dresler CM. Cytisine for smoking cessation: A research agenda. Drug and Alcohol Dependence. 2008;92(1-3):3-8.
83. Evins AE. Reassessing the safety of varenicline. The American journal of psychiatry. 2013;170(12):1385-7.
84. Faessel HM, Obach RS, Rollema H, Ravva P, Williams KE, Burstein AH. A review of the clinical pharmacokinetics and pharmacodynamics of Varenicline for smoking cessation. Clinical Pharmacokinetics. 2010;49(12):799-816.
85. Fagerstrom K. Varenicline can help smokers to stop smoking by gradual reduction. Evidence-Based Medicine. 2015;20(4):133.
86. Fagerstrom K, Balfour DJK. Neuropharmacology and potential efficacy of new treatments for tobacco dependence. Expert Opinion on Investigational Drugs. 2006;15(2):107-16.
87. Fagerstrom K, Hughes J. Varenicline in the treatment of tobacco dependence. Neuropsychiatric Disease and Treatment. 2008;4(2):353-63.
88. Feret B, Orr K. Varenicline: An oral partial nicotine agonist for smoking cessation. Formulary. 2006;41(6):265-72.
89. Fidler L, Anand A, Stanbrook M. Cardiovascular and neuropsychiatric risks of varenicline. The Lancet Respiratory Medicine. 2016;4(3):e9-e10.
90. Fillon M. Smoking Cessation Aids Alone Do Not Help Smokers Quit. CA Cancer Journal for Clinicians. 2018;68(6):389-91.
91. Freedman R. Searching for more effective smoking cessation treatment. American Journal of Psychiatry. 2013;170(8):818-20.
92. Frishman WH. Smoking cessation pharmacotherapy. Therapeutic Advances in Cardiovascular Disease. 2009;3(4):287-308.
93. Fujiwara H. Smoking is a disease and smokers are patients. Circulation Journal. 2010;74(4):628-9.
94. Gaballa D, Drowos J, Hennekens CH. Smoking Cessation: The Urgent Need for Increased Utilization of Varenicline. American Journal of Medicine. 2017;130(4):389-91.
95. Gershon AS, Campitelli MA, Hawken S, Sproule BA, Selby P. Reply to Goulden et al.: Risk of Bias in Study of Varenicline and Cardiovascular Outcomes. American journal of respiratory and critical care medicine. 2018;198(5):691-2.
96. Glover ED, Rath JM. Varenicline: Progress in smoking cessation treatment. Expert Opinion on Pharmacotherapy. 2007;8(11):1757-67.
97. Goldberg JF. Efficacy of varenicline for smoking cessation in bipolar disorder. Journal of Clinical Psychiatry. 2014;75(7):773-4.
98. Gomez-Coronado N, Walker AJ, Berk M, Dodd S. Current and Emerging Pharmacotherapies for Cessation of Tobacco Smoking. Pharmacotherapy. 2018;38(2):235-58.
99. Gonzales D, Rennard SI, Jorenby DE, Reeves KR. Comment: Oral varenicline for smoking cessation. The Annals of pharmacotherapy. 2007;41(4):720-1.
100. Gorelick DA. Sex difference in response to varenicline for smoking cessation. American Journal of Psychiatry. 2015;172(4):394-5.
101. Goulden R, Nguyen QD, Azoulay L. Risk of bias in study of varenicline and cardiovascular outcomes. American Journal of Respiratory and Critical Care Medicine. 2018;198(5):690-1.
102. Gunnell et al. Authors' reply. BMJ. 2009; 339.
103. Hajek P, McRobbie H, Myers K, Stapleton J, Dhanji AR. Is varenicline effectiveness declining in randomized trials? - Reply. Archives of Internal Medicine. 2011;171(19):1771-2.
104. Harrison-Woolrych M. Varenicline for smoking cessation. BMJ. 2012;345.
105. Harrison-Woolrych M. Mental health effects of varenicline Results from a new meta-analysis seem at odds with patients' real life experiences. BMJ. 2015;350 (h1168).
106. Hartmann-Boyce J, Aveyard P. Drugs for smoking cessation. BMJ. 2016;352 (i571).
107. Hays JT. Editorial: The risk-benefit balance of varenicline for smoking cessation. Journal of Thoracic Oncology. 2008;3(9):949-50.
108. Hays JT. Varenicline for smoking cessation: Is it a heartbreaker? Cmaj. 2011;183(12):1346-7.
109. Hays JT. Varenicline may reduce negative effect while aiding smoking cessation. Evidence-Based Medicine. 2014;19(1):23.
110. Hays JT, Ebbert JO. Adverse effects and tolerability of medications for the treatment of tobacco use and dependence. Drugs. 2010;70(18):2357-72.
111. Henningfield JE, Shiffman S, Ferguson SG, Gritz ER. Tobacco dependence and withdrawal: Science base, challenges and opportunities for pharmacotherapy. Pharmacology and Therapeutics. 2009;123(1):1-16.
112. Hillman T, Rajakulasingam K, Bhowmik A. Clinically significant outcomes in smoking cessation. Thorax. 2008;63(8):752; author reply -3.
113. Hilton JF, Prochaska JJ. Authors’ reply to Singh and Loke. BMJ : British Medical Journal. 2012;344:e4033.
114. Huber GL, Mahajan VK. Successful smoking cessation. Disease Management and Health Outcomes. 2008;16(5):335-43.
115. Ikram H, Zakir R, Haleem DJ, Choudhry AM. A combined therapy may be efficacious in ameliorating nicotine withdrawal symptoms. Medical Channel. 2013;19(3):7.
116. Jimenez-Ruiz C, Berlin I, Hering T. Varenicline: A novel pharmacotherapy for smoking cessation. Drugs. 2009;69(10):1319-38.
117. Jordan CJ, Xi ZX. Discovery and development of varenicline for smoking cessation. Expert Opinion on Drug Discovery. 2018;13(7):671-83.
118. Jorenby DE et al. In Reply. JAMA. 2006; 296.
119. Kalhan R, Wilkins JT, Hitsman BL. Tobacco smoking is a medical problem: We ought to treat it like one. American Journal of Respiratory and Critical Care Medicine. 2018;197(7):852-3.
120. Kapoor S. Varenicline and its role in smoking cessation. Preventive Cardiology. 2008;11(3):168-71.
121. Klesges RC, Johnson KC, Somes G. Varenicline for smoking cessation: Definite promise, but no panacea. Journal of the American Medical Association. 2006;296(1):94-5.
122. Kotz D, Van Schayck OCP. What justifies a placebo-controlled trial of varenicline for smoking cessation in patients with COPD? Chest. 2011;139(4):968-9.
123. Kotz D, Viechtbauer W, Simpson C, van Schayck OCP, West R, Sheikh A. Cardiovascular and neuropsychiatric risks of varenicline: Too good to be true? - Authors' reply. The Lancet Respiratory Medicine. 2015;3(12):e42.
124. Kotz D, Viechtbauer W, Simpson C, van Schayck OCP, West R, Sheikh A. Cardiovascular and neuropsychiatric risks of varenicline - Authors' reply. The Lancet Respiratory Medicine. 2016;4(3):e10.
125. Krebs P, Sherman SE. ACP Journal Club: review: varenicline for tobacco cessation does not increase CV serious adverse events. Annals of internal medicine. 2012;157(4):Jc2-.
126. Kress CM, Obi NU, Prochazka AV. In smokers with COPD, neither varenicline nor bupropion was linked to increased CV or neuropsychiatric risk vs NRT. Annals of Internal Medicine. 2017;167(6):JC31.
127. Lam CY, Minnix JA, Robinson JD, Cinciripini PM. A brief review of pharmacotherapies for smoking cessation. JNCCN Journal of the National Comprehensive Cancer Network. 2006;4(6):583-9.
128. Lam DC, Minna JD. How do we safely get people to stop smoking? Cancer prevention research (Philadelphia, Pa). 2011;4(11):1724-7.
129. Lam S, Patel PN. Varenicline: a selective alpha4beta2 nicotinic acetylcholine receptor partial agonist approved for smoking cessation. Cardiology in review. 2007;15(3):154-61.
130. Lamarche K. Review: varenicline, bupropion, and nicotine replacement therapies are effective for smoking cessation at 6 or 12 months. Evidence-based nursing. 2009;12(1):10.
131. Lancaster T, Cahill K. ACP Journal Club. Review: Varenicline does not differ from placebo for adverse neuropsychiatric events. Annals of internal medicine. 2015;163(2):Jc6.
132. Lasser KE, Boyd JW. Varenicline and smokers with mental illnesses. The Lancet. 2008;372(9645):1218-9.
133. Lavigne JE. Smoking cessation agents and suicide. BMJ (Online). 2009;339(7729):1042.
134. Le Foll B, George TP. Treatment of tobacco dependence: Integrating recent progress into practice. Cmaj. 2007;177(11):1373-80.
135. Le Houezec J, Aubin HJ. Pharmacotherapies and harm-reduction options for the treatment of tobacco dependence. Expert Opinion on Pharmacotherapy. 2013;14(14):1959-67.
136. Ledgerwood DM, Yskes R. Smoking cessation for people living with HIV/ AIDS: A literature review and synthesis. Nicotine and Tobacco Research. 2016;18(12):2177-84.
137. Leone FT, Schnoll R. Reframing the varenicline question: Have anecdotes and emotional filters clouded our decision making? The Lancet Respiratory Medicine. 2015;3(10):736-7.
138. Litt J. Varenicline and quitting. Australian Prescriber. 2008;31(5):115-6.
139. MacSuibhne S, Giwa TA, McCauley MD. Varenicline (champix) - associated manic relapse in bipolar affective disorder. Irish Medical Journal. 2010;103(9):1.
140. Madara J, West FM, Zappetti D. What's the Risk? Varenicline May Increase Cardiovascular Complications in Users. Clinical Pulmonary Medicine. 2018;25(6):233.
141. Mahvan T, Namdar R, Voorhees K, Smith PC, Flake D. Which smoking cessation interventions work best? Journal of Family Practice. 2011;60(7):430-1.
142. McIntyre RS. Varenicline and suicidality: A new era in medication safety surveillance. Expert Opinion on Drug Safety. 2008;7(5):511-4.
143. McMahon LR. Green tobacco sickness: mecamylamine, varenicline, and nicotine vaccine as clinical research tools and potential therapeutics. Expert Review of Clinical Pharmacology. 2019.
144. Mendelsohn C. Recent developments in smoking cessation. Medicine Today. 2008;9(12):38-45.
145. Mendelsohn CP, Kirby DP, Castle DJ. Smoking and mental illness. An update for psychiatrists. Australasian Psychiatry. 2015;23(1):37-43.
146. Moore TJ, Furberg CD. Risk of psychiatric side effects with varenicline. BMJ. 2009;339(7733):1272.
147. Nahvi S, Cooperman NA. Review: The need for smoking cessation among HIV-positive smokers. AIDS Education and Prevention. 2009;21(SUPPL. 3):14-27.
148. Niaura R. Varenicline and suicide: Reconsidered and reconciled. Nicotine and Tobacco Research. 2016;18(1):1.
149. Niaura R, Chander G, Hutton H, Stanton C. Interventions to address chronic disease and HIV: Strategies to promote smoking cessation among HIV-infected individuals. Current HIV/AIDS Reports. 2012;9(4):375-84.
150. Nides M. Update on Pharmacologic Options for Smoking Cessation Treatment. American Journal of Medicine. 2008;121(4 SUPPL.):S20-S31.
151. Nielen JTH, Burden AM. Cardiovascular and neuropsychiatric risks of varenicline: Too good to be true? The Lancet Respiratory Medicine. 2015;3(12):e38-e9.
152. O'Brien CP. Varenicline as maintenance therapy. Current psychiatry reports. 2007;9(5):347-8.
153. O'Malley SS. Varenicline and the evaluation of neuropsychiatric adverse events in smokers. Biological Psychiatry. 2011;69(11):1017-8.
154. Ockene I, Salmoirago-Blotcher E. Varenicline for smoking cessation in patients with coronary heart disease. Circulation. 2010;121(2):188-90.
155. Odeyale F, Udo I. Cardiovascular and neuropsychiatric risks of varenicline: Too good to be true? The Lancet Respiratory Medicine. 2015;3(12):e38.
156. Potter AS. Smoking cessation in men and women. American Journal of Psychiatry. 2014;171(11):1148-50.
157. Preziosi P. Faster drug approval: challenges for safety. Expert Opinion on Drug Safety. 2016;15(9):1205-18.
158. Primack BA. Varenicline, combination NRT, and nicotine patch did not differ for smoking cessation at 6 mo: Commentary. Annals of Internal Medicine. 2016;164(10):JC54.
159. Prochaska JJ. Quitting smoking is associated with long term improvements in mood. BMJ. 2014;348 (g1562).
160. Prochaska JJ. Commentary on Roberts et al. (2016): Bupropion and varenicline are efficacious and well-tolerated cessation medications for smokers with serious mental illness. Addiction . 2016;111(4):613-4.
161. Prochaska JJ, Benowitz NL. The Past, Present, and Future of Nicotine Addiction Therapy. Annual review of medicine. 2016;67:467-86.
162. Prochazka AV. Varenicline increased smoking cessation at 24 weeks in patients hospitalized with ACS and motivated to quit. Annals of Internal Medicine. 2016;164(8):JC43.
163. Prochazka AV, Caverly TJ. ACP Journal Club. Review: varenicline is better than bupropion but not nicotine patch for smoking abstinence in adults. Annals of internal medicine. 2012;157(6):Jc3-7.
164. Ratchford EV, Black IJH. Approach to smoking cessation in the patient with vascular disease. Current Treatment Options in Cardiovascular Medicine. 2011;13(2):91-102.
165. Ratchford EV, Evans NS. Smoking cessation. Vascular Medicine. 2016;21(5):477-9.
166. Reid RD, Mullen KA, Pipe AL. Systematic approaches to smoking cessation in the cardiac setting. Current opinion in cardiology. 2011;26(5):443-8.
167. Reid RD, Mullen KA, Pipe AL. Tackling smoking cessation systematically among inpatients with heart disease. Cmaj. 2018;190(12):E345-E6.
168. Reiner Z. The importance of smoking cessation in patients with coronary heart disease. International Journal of Cardiology. 2018;258:26-7.
169. Rennard SI. Introduction. American Journal of Medicine. 2008;121(4 SUPPL.):S1-S2.
170. Rigotti NA. Cytisine - A tobacco treatment hiding in plain sight. New England Journal of Medicine. 2014;371(25):2429-30.
171. Rigotti NA. Improving the success of treating tobacco smokers. JAMA Internal Medicine. 2015;175(2):272-3.
172. Robey RB, Block CA, O'Rourke DJ. Varenicline for smoking reduction prior to cessation. Jama. 2015;313(22):2285.
173. Robson N. Varenicline: A new pharmacotherapy for smoking cessation in primary care practice. South African Family Practice. 2011;53(3):217-22.
174. Rollema H, Faessel HM. Pharmacodynamic and pharmacokinetic profiles of the alpha4beta2 nicotinic acetylcholine receptor partial agonist varenicline, a smoking cessation aid. Journal of Pediatric Biochemistry. 2010;1(2):175-84.
175. Rose JE. Pre-cessation varenicline treatment vs post-cessation NRT: An uneven playing field. Thorax. 2008;63(8):751-2.
176. Rose JE, Behm FM. Response to Gorelick. Am J Psychiatry.2015.
177. Rowe SL, Daugherty KK. A targeted review of smoking cessation aids and their pharmacology for the pharmacy technician. Journal of Pharmacy Technology. 2010;26(5):300-4.
178. Rowland K. Review: Nicotine replacement therapy increases CVD events; Bupropion and varenicline do not. Annals of Internal Medicine. 2014;160(8):JC2.
179. Rudorfer MV, Hillefors M. Assessing psychiatric adverse effects during clinical drug development. Pharmaceutical Medicine. 2012;26(6):363-94.
180. Russo P, Cesario A, Rutella S, Veronesi G, Spaggiari L, Galetta D, et al. Impact of genetic variability in nicotinic acetylcholine receptors on nicotine addiction and smoking cessation treatment. Current Medicinal Chemistry. 2011;18(1):91-112.
181. Rutter JL. Symbiotic relationship of pharmacogenetics and drugs of abuse. AAPS Journal. 2006;8(1):E174-E84.
182. Samet JM. Smoking cessation: Benefits versus risks of using pharmacotherapy to quit. Circulation. 2014;129(1):8-10.
183. Samuels L. Varenicline: Cardiovascular safety. CMAJ. 2011;183(12):1407-8.
184. Scherman A, Tolosa JE, McEvoy C. Smoking cessation in pregnancy: a continuing challenge in the United States. Therapeutic Advances in Drug Safety. 2018;9(8):457-74.
185. Schroeder SA. How clinicians can help smokers to quit. JAMA - Journal of the American Medical Association. 2012;308(15):1586-7.
186. Schroeder SA, Koh HK. Tobacco control 50 years after the 1964 surgeon general's report. JAMA. 2014;311(2):141-3.
187. Schroeder SA, Sox HC. Trials that matter: Varenicline: A designer drug to help smokers quit. Annals of Internal Medicine. 2006;145(10):784-5.
188. Sharma A, Thakar S, Lavie CJ, Garg J, Krishnamoorthy P, Sochor O, et al. Cardiovascular adverse events associated with smoking-cessation pharmacotherapies. Current cardiology reports. 2015;17(1):554.
189. Simon JA. Smoking cessation interventions: a primer for physicians: Comment on "Use of varenicline for 4 weeks before quitting smoking". Archives of internal medicine. 2011;171(8):777-8.
190. Singh S. Varenicline: Quantifying the risk. Cmaj. 2011;183(12):1405-7.
191. Singh S, Loke YK. Flaws in analysis lead to misleading conclusions about varenicline's safety in smoking cessation. BMJ. 2012;344 (7861) (no pagination)(e3873).
192. Singh S, Loke YK, Spangler JG, Furberg CD. Authors’ response. Canadian Medical Association Journal. 2011;183(12):1408-.
193. Sofuoglu M, Duffey D, Mooney ME. Varenicline increases smoking abstinence at 6 months to a year compared with placebo or bupropion; nausea is the most commonly reported adverse effect. Evidence-Based Medicine. 2011;16(4):113-4.
194. Sorbera LA, Castaner J. Varenicline tartrate. Drugs of the Future. 2006;31(2):117-22.
195. Sotiriou I, Chalkiadaki K, Nikolaidis C, Sidiropoulou K, Chatzaki E. Pharmacotherapy in smoking cessation: Corticotropin Releasing Factor receptors as emerging intervention targets. Neuropeptides. 2017;63:49-57.
196. Spangler JG. Comment and reply: A double-blind study evaluating the long-term safety of varenicline for smoking cessation. Current Medical Research and Opinion. 2008;24(2):577-8.
197. Spiegler P. Going straight to the source: A new tool for smoking cessation. Clinical Pulmonary Medicine. 2006;13(6):366-7.
198. Squire EN. Varenicline: Quantifying the risk. Cmaj. 2011;183(12):1404-5.
199. Stapleton J. Do the 10 UK suicides among those taking the smoking cessation drug varenicline suggest a causal link? Addiction. 2009;104(5):864-5.
200. Stephens LA, Stevermer JJ. PURLs: Counseling is a must with this smoking cessation aid. The Journal of family practice. 2012;61(3):156-76.
201. Suchanek Hudmon K, Corelli RL, Prokhorov AV. Current approaches to pharmacotherapy for smoking cessation. Therapeutic Advances in Respiratory Disease. 2010;4(1):35-47.
202. Sutton S. Smoking cessation. Medicine. 2008;36(4):226-9.
203. Takagi H, Umemoto T. Varenicline: quantifying the risk. CMAJ. 2011;183(12):1404; author reply 5, 7.
204. Tavares Jr AR. Varenicline for tobacco dependence. The New England journal of medicine. 2009;360(7):731.
205. Thomas KH, Martin RM, Davies NM, Metcalfe C, Windmeijer F, Gunnell D. Authors' reply to Davies. BMJ. 2013;347 (no pagination)(f7068).
206. Tonstad S, Rollema H. Varenicline in smoking cessation. Expert Review of Respiratory Medicine. 2010;4(3):291-9.
207. Valenca MM. Bath-related headache induced by varenicline. Arquivos de neuro-psiquiatria. 2012;70(11):908.
208. Walker N, Howe C, Glover M, McRobbie H, Barnes J, Nosa V, et al. Cytisine versus nicotine for smoking cessation. Obstetrical and Gynecological Survey. 2015;70(4):255-6.
209. Walsh RA. Australia's experience with varenicline: usage, costs and adverse reactions. Addiction. 2011;106(2):451-2.
210. Ware JJ, Davies NM, Munafo MR. Importance of national context in the translation of personalised treatments for smoking cessation. The Lancet Respiratory Medicine. 2015;3(2):91-3.
211. Weinberger AH. Smoking cessation and adults with serious mental illness: The Need for More Research at Every Step of the Quit Process. Nicotine and Tobacco Research. 2016;18(3):227-8.
212. West R. The clinical significance of 'small' effects of smoking cessation treatments. Addiction. 2007;102(4):506-9.
213. Williams JM. Varenicline should be used as a first-line treatment to help smokers with mental illness quit. Journal of Dual Diagnosis. 2012;8(2):113-6.
214. Williams JM, Steinberg MB, Steinberg ML, Gandhi KK, Ulpe R, Foulds J. Varenicline for tobacco dependence: Panacea or plight? Expert Opinion on Pharmacotherapy. 2011;12(11):1799-812.
215. Williams KE et al. Authors’ reply. Current Medical Research and Opinion. 2008; 24.
216. Wise L, Parkinson J, Raine J, Breckenridge A. New approaches to drug safety: A pharmacovigilance tool kit. Nature Reviews Drug Discovery. 2009;8(10):779-82.
217. Woods DJ, Caswell MD. Varenicline: quantifying the risk. CMAJ. 2011;183(12):1404; author reply 5, 7.
218. Wu BS, George TP. Pharmacotherapies for nicotine addiction in adults and adolescents. Journal of Pediatric Biochemistry. 2010;1(2):165-74.
219. Wu J, Sin DD. Improved patient outcome with smoking cessation: When is it too late? International Journal of COPD. 2011;6(1):259-67.
220. Yardley MM, Mirbaba MM, Ray LA. Pharmacological Options for Smoking Cessation in Heavy-Drinking Smokers. CNS Drugs. 2015;29(10):833-45.
221. Zaparoli JX, Galduroz JCF. Varenicline: The risks of over-the-counter sales in Brazil. Revista Brasileira de Psiquiatria. 2013;35(4):442-3.
222. Zawertailo L. Safety of smoking cessation drugs for mentally ill patients. The Lancet. 2016;387(10037):2481-2.
