# Supplementary material for: Association Between Conflicts of Interest and Authors’ Positions on Harms of Varenicline: a Cross-Sectional Analysis
Source: J Gen Intern Med. 2021 May 26;37(2):290–7. doi: 10.1007/s11606-021-06915-1 (PMC8811060; doi:10.1007/s11606-021-06915-1)
Supplement: Supplementary file 4 — (DOCX 22 kb) [file 11606_2021_6915_MOESM4_ESM.docx]

**Supplementary File 4.**

**Table S1. Sensitivity analysis on the association between financial conflicts of interest and author’s position on cardiovascular risk, psychiatric risk, and overall position on varenicline**

|  |  | **Excluding articles with insufficient information and unclear positions** | | |
| --- | --- | --- | --- | --- |
|  |  | N | OR | 95% CI |
| **Cardiovascular safety concerns** |  | 51 | 6.67 | 1.32-33.75 |
| **Psychiatric safety concerns** |  | 96 | 17.32 | 5.75-52.20 |
| **Overall position on varenicline** |  | 152 | 35.33 | 4.66-267.67 |

OR: odds ratio; CI: confidence interval

**Table S2. Post-hoc sensitivity analysis for association between financial conflicts of interest and author’s position on cardiovascular safety concerns adjusted for publication year (published before and after the first FDA safety advisory on cardiovascular risks)**

|  | **Cardiovascular safety concerns** | |
| --- | --- | --- |
|  | **OR** | **95% CI** |
| **Crude model (N=67)** | | |
| **Conflicts of interest or pharmaceutical funding** |  |  |
| COI^*^ | 4.00 | 1.32-12.16 |
| No COI^**^ | 1.00 | - |
| **Adjusted (N=67)** |  |  |
| **Conflicts of interest or pharmaceutical funding** |  |  |
| COI^*^ | 4.09 | 1.34-12.54 |
| No COI^**^ | 1.00 |  |
|  |  |  |

COI: conflicts of interest; OR: odds ratio; CI: confidence interval

^*^Includes articles disclosing pharmaceutical industry funding (alone or with other organisations), authors’ conflicts of interest with pharmaceutical companies, or both.

**Includes articles with no COI, no pharmaceutical funding, and no COI disclosure.

**Table S3. Post-hoc sensitivity analysis for association between financial conflicts of interest and author’s position on psychiatric safety concerns adjusted for publication year (published before and after the FDA removal of the Boxed Warning for varenicline’s psychiatric risks)**

|  | **Psychiatric safety concerns** | |
| --- | --- | --- |
|  | **OR** | **95% CI** |
| **Crude model (N=131)** | | |
| **Conflicts of interest or pharmaceutical funding** |  |  |
| COI^*^ | 8.51 | 3.79-19.11 |
| No COI^**^ | 1.00 | - |
| **Adjusted (N=131)** |  |  |
| **Conflicts of interest or pharmaceutical funding** |  |  |
| COI^*^ | 10.52 | 4.40-25.16 |
| No COI^**^ | 1.00 |  |
|  |  |  |

COI: conflicts of interest; OR: odds ratio; CI: confidence interval

^*^Includes articles disclosing pharmaceutical industry funding (alone or with other organisations), authors’ conflicts of interest with pharmaceutical companies, or both.

**Includes articles with no COI, no pharmaceutical funding, and no COI disclosure.

**Table S4. Post-hoc sub-group analysis stratified by type of article**

|  | **Letters** | | |  | **Other articles^*^** | | |
| --- | --- | --- | --- | --- | --- | --- | --- |
|  | N | OR | 95% CI |  | N | OR | 95% CI |
| **Cardiovascular safety concerns** | 21 | 2.29 | 0.32-16.51 |  | 46 | 5.18 | 1.34-20.06 |
| **Psychiatric safety concerns** | 26 | 24.00 | 1.95-295.06 |  | 105 | 7.14 | 2.98-17.11 |
| **Overall position on varenicline** | 53 | 12.00 | 3.07-46.88 |  | 168 | 7.86 | 3.41-18.11 |

OR: odds ratio; CI: confidence interval

^*^Includes narrative reviews, editorials, commentaries, viewpoints, bulletin articles, and other types of opinion pieces.

**Table S5.** **Altmetric score between articles with and without conflict of interest by publication year**

|  | **Altmetric score by publication year** | | | | | | | | |
| --- | --- | --- | --- | --- | --- | --- | --- | --- | --- |
|  | **2011** | **2012** | **2013** | **2014** | **2015** | **2016** | **2017** | **2018** | **2019** |
| **COI^*^** | | | | | | | | | |
| N | 7 | 5 | 4 | 5 | 7 | 6 | 2 | 4 | 1 |
| Mean | 0.1 | 1 | 6.8 | 25.2 | 2.6 | 40 | 35 | 40 | 2 |
| Median | 0 | 1 | 7.5 | 5 | 2 | 12.5 | 35 | 18 | 2 |
| **No COI^**^** | | | | | | | | | |
| N | 10 | 11 | 5 | 9 | 5 | 8 | 4 | 6 | 1 |
| Mean | 2.7 | 3.2 | 0.6 | 60.1 | 6.2 | 1.5 | 10 | 2.2 | 0 |
| Median | 0 | 1 | 1 | 2 | 2 | 1 | 4 | 1 | 0 |
| **P-value** | 0.36 | 0.44 | 0.13 | 0.74 | 0.57 | 0.00 | 0.16 | 0.13 | 0.32 |

COI: conflicts of interest

^*^Includes articles disclosing pharmaceutical industry funding (alone or with other organisations), authors’ conflicts of interest with pharmaceutical companies, or both.

^**^Includes articles with no COI, no pharmaceutical funding, and no COI disclosure.
